# Circulating miRNA-202-3p is a potential novel biomarker for diagnosis of type 1 gastric neuroendocrine neoplasms

**DOI:** 10.1186/s12876-021-01769-7

**Published:** 2021-04-23

**Authors:** Dou Dou, Xiao-kou Li, Qi-sheng Xia, Ying-ying Chen, Yuan-liang Li, Chao Wang, Zhi-rong Qi, Huang-ying Tan

**Affiliations:** 1 Beijing University of Chinese Medicine, Beijing, China; 2 Department of Integrative Oncology, China-Japan Friendship Hospital, Beijing, China; 3 Institute of Clinical Medical Sciences, China-Japan Friendship Hospital, Beijing, China

**Keywords:** MicroRNA-202-3p, Biomarker, Type 1 gastric neuroendocrine neoplasms

## Abstract

**Background:**

Currently, there are no circulating diagnostic biomarkers for gastric neuroendocrine neoplasms (g-NENs). In previous studies, we found that miRNA-202-3p is overexpressed in the tumour tissue of type 1 g-NEN. We speculated that miRNA-202-3p is also likely to be highly expressed in circulating blood.

**Methods:**

A total of 27 patients with type 1 g-NEN and 27 age- and sex-matched control participants were enrolled in this study. The miRNA-202-3p levels in serum obtained from the participants were measured by qRT‐PCR. The expression level of miRNA-202-3p in the samples was calculated by comparison with a standard curve.

**Results:**

The clinical characteristics of the patients were similar to those of the patient samples in previous reports. Expression of miRNA-202-3p was significantly higher in the patient group (3.84 × 10^7^ copies/nl) than in the control group (0.635 × 10^7^ copies/nl). The area under the ROC curve (AUC) was 0.878 (95% CI: 0.788–0.968), and the optimal cut-off point was approximately 1.12 × 10^7^ copies/nl. The sensitivity and specificity were 88.9% and 77.8%, respectively.

**Conclusion:**

This study suggests that miRNA-202-3p is potentially useful as a biomarker of type 1 g-NEN; further investigation and verification should be performed in future research.

## Background

Gastric neuroendocrine neoplasms (g-NENs) are rare malignancies that are mainly derived from enterochromaffin-like (ECL) cells [1]. According to the WHO classification, well-differentiated g-NENs derived from ECL cells can be divided into types 1, 2 and 3 [2, 3]. Among them, type 1 g-NEN is related to chronic atrophic gastritis with hypergastrinemia and achlorhydria.

Due to the lack of specific symptoms of NENs, circulating biomarkers can be of significant diagnostic value. At present, serum chromogranin A (CgA) is the biomarker that is most widely used to determine tumour burden and to monitor response to treatment in patients with NENs. Unfortunately, CgA has a relatively high false negative rate and is poorly associated with disease severity [4].

MicroRNAs (miRNAs) are small, non-coding RNAs that participate in posttranscriptional regulation either by repressing mRNA translation or by promoting mRNA degradation [5]. Dysregulated miRNAs have been linked to various tumours and can play pro-oncogenic and anti-oncogenic roles in different diseases [6, 7]. Circulating miRNAs, which are present in cell-free body fluids such as urine, saliva, plasma and serum, have been gaining attention as tumour biomarkers in recent years. The levels of serum miRNAs have been shown in many studies to differ in NEN patients and control participants. Some miRNAs were reported to be upregulated (e.g., miRNA-200a, miRNA-21-5p and miRNA-22-3p in small bowel NENs [8, 9]), while others were shown to be downregulated (e.g., miR-150-5p in small bowel NENs [9] and miR-886-3p in small cell lung cancer (SCLC) [10]). However, studies focusing on miRNAs in patients with type 1 g-NEN are rare. The only published article on this subject investigated miR-222 but did not discuss its potential diagnostic value based on area under the ROC curve (AUC), sensitivity or specificity [11].

In previous studies [12], we found that miRNA-202-3p is overexpressed in the tumour tissue of type 1 g-NEN. Considering that type 1 g-NEN tumour tissue is rich in blood supply [13], the aim of this study was to determine whether miRNA-202-3p is highly expressed in the circulating blood of patients with type 1 g-NEN.

## Materials and methods

### Materials

A cohort of 27 patients who had been diagnosed with type 1 g-NEN and hospitalized at the Department of Integrative Oncology of the China-Japan Friendship Hospital between January 2018 and January 2019 and 27 age- and sex-matched control participants were enrolled in this study. The inclusion criteria for type 1 g-NEN patients were based on the 2016 NCCN guidelines [14]. Briefly, they were as follows: (1) a clear pathological diagnosis of a well-differentiated g-NEN and (2) presence of all of the following clinical characteristics of type 1 g-NEN: hypergastrinemia, achlorhydria, and chronic atrophic gastritis. A 2-ml blood sample was obtained from each patient and from each control participant. The samples were centrifuged (5000 rpm for 15 min at 4 °C), and the serum was extracted, transferred to RNase‐free tubes, and stored at − 80 °C for the following experiments. To investigate the characteristics of the patients in more detail, gastroscopy and pathology were performed after the collection of blood samples. Specifically, if a polyp was found by gastroscopy, biopsy and pathological examination were performed. However, since patients were included in the study during their follow-up, some patients had no tumours in the stomachs (which had been removed during the previous treatment). In this situation, two pieces of mucosa were taken from the stomach body for pathological examination.

### Total RNA extraction

Total RNA was extracted from the serum samples using the miRNeasy Serum/Plasma Kit (Qiagen, Germany). Each 200-μl serum sample was thoroughly mixed with 1 ml QIAzol, and the mixture was incubated for 5 min at room temperature. Then, 200 μl of chloroform was added, the sample was vigorously shaken by hand for 15 s, and the mixture was centrifuged at 12,000 rpm for 15 min at 4 °C. The supernatant was transferred to a new tube, and 1.5 volumes of 100% ethanol were added to the tube. After mixing, the samples were transferred to an RNeasy MinElute spin column and centrifuged at 8000 rpm for 15 s at 4 °C. The RNeasy MinElute spin column was washed sequentially with Buffer RWT, Buffer RPE and 75% ethanol. The sample was then centrifuged at maximum speed for 5 min at 4 °C. Finally, the RNA pellet was dissolved in 14 μl DEPC-treated water. RNA quality was measured using the NanoDrop One (Thermo Fisher Scientific, USA).

### cDNA synthesis

Reverse transcription was performed using the TaqMan® Advanced miRNA cDNA Synthesis Kit. All procedures were performed according to the TaqMan® Advanced miRNA Assays User Guide. First, the miRNA molecules were polyadenylated by poly(A) polymerase. Second, the adaptor ligation reaction was performed. Third, the miRNA molecules were reverse-transcribed into cDNA using oligo-dT primers and reverse transcriptase (RT). Finally, 5 µL of the RT reaction product was used to perform the miR-Amp reaction.

### Quantitative real-time PCR

When the RT reactions had been completed, quantitative real-time PCR (qRT-PCR) was performed using the TaqMan® Advanced miRNA Assay Kit (Thermo Fisher Scientific, USA). A 1:10 dilution of the cDNA template was prepared. For a 20-µL reaction, 10 µL of TaqMan® Fast Advanced Master Mix (2X), 1 µL of TaqMan® Advanced miRNA Assay (20X), 4 µL of RNase-free water and 5 µL of the diluted cDNA template were combined. PCRs were conducted in a Quant Studio 5 (Thermo Fisher Scientific, USA). The reactions were incubated in a 96-well plate at 95 °C for 20 s, followed by 40 cycles of 95 °C for 1 s and 60 °C for 20 s. RT-PCR was performed in triplicate, and the average values after discarding any outliers (> 2 standard deviations) were used in the subsequent analyses.

### Standard curve method

The standard curve method was used to obtain absolute values in PCR. MiRNA-202-3p (5′-AGAGGTATAGGGCATGGGAA-3′) was synthesized (Gene Pharma, Shanghai) and a known concentration of this miRNA was serially diluted fourfold to generate standard solutions at 6 different concentrations. The concentration and the number of molecules of the six gradient standards were calculated as shown in the following table:

**Table:** 

Standard	Concentration (pmol/L)	Molecules (copies/nl)
S1	500	600,000,000
S2	125	150,000,000
S3	31.25	37,500,000
S4	7.81	9,375,000
S5	1.95	2,343,750
S6	0.49	585,937.5

The six gradient standards were reverse-transcribed at the same time as the samples, and PCR was performed in the same 96-well plate. Finally, the number of molecules in each sample was calculated by comparing the ΔCt value with the standard curve. According to the dilution procedure, the conversion formula for the final unit was as follows: copy number = absolute quantitative PCR × (total RNA concentration/5)/2; the units were copies/nl.

### Statistics

Each experimental data point was obtained from three technical replicates. Continuous data are represented as median quartile, and classification data are represented as the percentage of use cases. The rank sum test and the chi-square test were used to compare differences between groups. Receiver operating characteristic (ROC) curves and AUCs were determined to assess the diagnostic ability of miRNA-202-3p in type 1 g-NEN, and the cut-off value was calculated using the Jordan index. SPSS 20.0 software (SPSS, Chicago, IL) was used to perform the statistical analyses, and *p* < 0.05 was considered statistically significant. The graphs were plotted by GraphPad Prism V.5 software.

## Results

### Clinicopathological characteristics of the study population

The baseline characteristics of the type 1 g-NEN patients and the control participants in this study are listed in Tables 1, 2 and 3. Of the 27 patients, 6 were male (22.2%) and 21 were female (77.8%), and the average age at diagnosis was 56 years (52 for men, 63 for women; age range, 27–78). Of the 27 control participants, 6 were male (22.2%) and 21 were female (77.8%), and the average age was 52 years (50 for men, 57 for women; age range, 45–67). There were no significant differences between the ages of the type 1 g-NEN patients and those of the control participants.

**Table 1:** Clinical characteristics of type 1 g-NEN patients and control participants

Variable	Patient group (n = 27)	Control group (n = 27)	*p*
Male/female (n/n)	6/21	6/21	–
Age (years)	56 (52, 63)	52 (50, 57)	0.191

**Table 2:** Patient condition at the time of diagnosis

Variable	Patient group (n = 27)	Rate (%)	X ± SE
Tumour grading			–
G1	26	96.3	–
G2	1	3.7	–
CgA (ng/ml)			215.19 ± 53.63
> 100	10	55.6 (n = 18)	–
< 100	8	44.4 (n = 18)	–
Unknown	9	–	–
Gastrin (pg/ml)			796.48 ± 99.47
> 100	27	100	–
< 100	0	0	–

**Table 3:** Endoscopic manifestations and pathological results for 27 patients

Variable	Patient group (n = 27)	Rate (%)
Tumour site		
Gastric fundus and/or body	22	81.5
Gastric antrum	0	0
No polyps	5	18.5
Tumour number		
Single tumour	4	14.8
Multiple tumours	18	66.7
No polyps	5	18.5
Maximum tumour diameter (cm)		
< 1	19	70.3
1–2	3	11.1
> 2	0	0
No polyps	5	18.5
Depth of tumour invasion		
Mucosal layer	20	74.1
Submucosal layer	2	7.4
No polyps	5	18.5
Tumour morphology		
Polyp or protuberance	22	81.5
No polyps	5	18.5
Pathological results		
Chronic inflammation	6	22.2
Hyperplasia	11	40.7
Dysplasia	2	7.4
Tumour	8	29.6
OLGIM stage		
Stage 0	18	66.7
Stage I	6	22.2
Stage II	3	11.1
Stage III	0	0
Stage IV	0	0

Regarding the patients' condition at the time of diagnosis (Table 2), the pathological grades were tumour G1 (96.3%, 26/27) and G2 (3.7%, 1/27), all patients had a serum gastrin levels higher than the reference range (100 pg/ml), 55.6% (10/18) of the patients had serum CgA levels higher than the reference range (100 ng/ml).

For endoscopic features this time (Table 3), the main manifestations were polyps or protuberances of the fundus and/or body of the stomach (81.5%, 22/27); 66.7% (18/27) of the patients had multiple lesions, and 70.3% (19/27) had tumour less than 1 cm in diameter. The endoscopic ultrasound investigation results showed that the lesions were mainly located in the mucosa (74.1%, 20/27), and the pathological results indicated chronic inflammation (22.2%, 6/27), hyperplasia (40.7%, 11/27), dysplasia (7.4%, 2/27) and tumour (29.6%, 8/27). OLGIM (operative link for gastric intestinal metaplasia assessment) stages 0, I, and II, but not III or IV, were represented among the patients.

### MiRNA-202-3p levels in the sera of control participants and patients with type 1 g-NEN

**Fig. 1:**
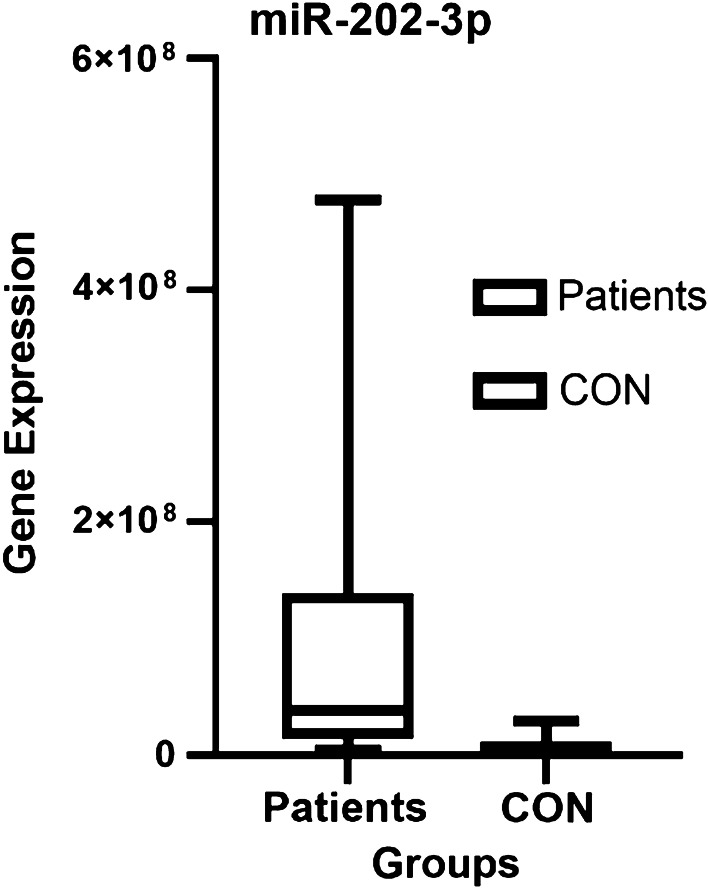
Expression of miRNA in serum from type 1 g-NEN patients and control participants. *Patients* type 1 g-NEN patients; *CON* control participants

We quantified the expression of miRNA-202-3p in the serum of 27 control participants and 27 patients with type 1 g-NEN. The expression levels of miRNA-202-3p in serum are listed in Table 4. As shown in Fig. 1, expression of miRNA-202-3p was significantly higher (*p* < 0.001) in the patient group (3.84 × 10^7^ copies/nl (range 1.36 × 10^7^–14 × 10^7^ copies/nl)) than in the control group (0.635 × 10^7^ copies/nl (range 0.297 × 10^7^–1.09 × 10^7^ copies/nl)).

**Table 4:** miRNA-202-3p levels in the sera of control participants and in patients with type 1 g-NEN

miRNA-202-3p	Patient group (copies/nl)	Control group (copies/nl)	*p*
Mean	9.22 × 10^7^	0.867 × 10^7^	0.000
Median	3.84 × 10^7^	0.635 × 10^7^

### Diagnostic specificity and sensitivity of miRNA-202-3p in type 1 g-NEN

**Fig. 2:**
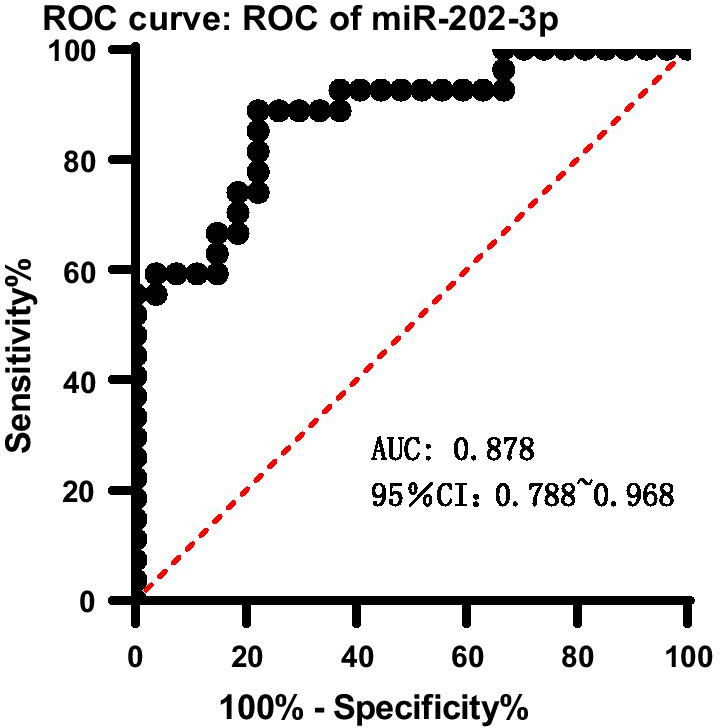
Receiver operating characteristic curves for the diagnostic accuracy of miRNA-202-3p

To further explore the suitability of using miRNA-202-3p as a potential diagnostic biomarker for type 1 g-NEN, we performed ROC curve analysis. The results indicated that miR‐202‐3p showed moderate power to differentiate type 1 g-NEN patients from control participants (Fig. 2). The AUC was 0.878 (Fig. 2). The 95% confidence interval for miRNA-202-3p was 0.788 ~ 0.968, the optimal cut-off point was approximately 1.12 × 10^7^ copies/nl, and the sensitivity and specificity were 88.9% and 77.8%, respectively, as shown in Table 5.

**Table 5:** Characteristics of miRNA-202-3p expression in type 1 g-NEN patients based on the optimal cut-off point

miRNA-202-3p	Cut-off point (copies/nl)	Sensitivity	Specificity	Youden index
	1.12 × 10^7^	0.889	0. 778	0.667

## Discussion

Type 1 g-NEN has long been considered a rare disease, and few studies have been performed. However, in recent years, the incidence of all NENs has increased sharply due to increased diagnostic testing. The annual age-adjusted incidence of all NENs was 1.09 per 100,000 persons in 1973, and it had increased to 6.98 per 100,000 persons by 2012 [15]. The proportion of g-NENs among all NENs in the United States, Austria and Mainland China was 6% [16], 23% [17], and 29.6% [18], respectively. Type 1 g-NEN is the most common type of g-NEN and accounts for approximately 35.7% of g-NENs [2].

Type 1 g-NEN arises in patients who have autoimmune atrophic gastritis, which causes atrophy and reduces the number of acid-secreting gastric parietal cells. This leads to achlorhydria and corresponding symptoms such as bloating, indigestion, and constipation [19]. Nevertheless, most patients with type 1 g-NEN lack typical symptoms, and a large number of them have no obvious clinical symptoms [20–22]. For this reason, type 1 g-NEN is often accidentally discovered during gastroscopy. Gastroscopic examinations are invasive and have not been widely popularized. Therefore, there is an urgent need to develop novel non-invasive tests for type 1 g-NEN.

The results of this study showed that serum miRNA-202-3p levels were significantly elevated in type 1 g-NEN patients compared to control participants (*p* < 0.001). The AUC was 0.878 (95% CI: 0.788 ~ 0.968), indicating that serum miRNA-202-3p level may be a potential biomarker for type 1 g-NEN. Analysis of the results showed that the gastroscopic findings for the patients in this study were consistent with the typical manifestation of type 1 g-NEN. Most patients had multiple tumours less than 1 cm in size, and the pathology grade was G1 or G2. However, serum miRNA-202-3p levels in patients with different pathologies and different gastroscopic findings, including polyp size, number of polyps and OLGIM state, had no significant stratified diagnostic value. This may be due to the limited number of samples.

MiRNA-202 is located at 10q26 (position 135,061,015 -135,061,124 on chromosome 10), and its mature single-stranded miRNA sequence is 5′-UUCCUAUGCAUAUACUUCUUUG-3′ [23]. Although miRNA-202 belongs to the let-7 family, a well-known cancer-suppressing family, in recent years some studies have reported that it has carcinogenic potential in certain types of tumours such as endometrial cell proliferation, multiple myeloma, prostate cancer, breast cancer and intestinal gastric carcinoma [24–28]. In our previous studies, miRNA-202 was also found to be upregulated in type 1 g-NEN [12].

Circulating miRNAs can be detected in serum and in other body fluids and are used as biomarkers of many kinds of tumours [28–31]. The stability of miRNAs in clinical serum samples raises important and intriguing questions regarding the mechanism by which miRNAs are protected from endogenous RNase activity. One hypothesis is that some miRNAs are packaged inside exosomes that are secreted from cells. MiRNA-202-3p, which is highly expressed in type 1 g-NEN tumour tissue, may enter the blood through exosomes in this manner. In addition, because type 1 g-NEN is an endocrine tumour, its tumour tissue has a typical endocrine organ-like structure with a dense vascular network and abundant blood flow [13]. It can be speculated that this is one of the causes of elevated serum miRNA-202-3p levels in patients with type 1 g-NEN. Other possible explanations include protection via association with other molecules (RNA–protein complex such as AGO2) or modifications of the miRNAs that make them resistant to RNase activity [32]. Additional studies that explore these hypotheses are needed.

Technological advancements have made it possible to quantify miRNAs with accuracies comparable to those for more traditional RNAs [33]. In this study, absolute (standard curve) quantification of RT-PCR products was used to measure the expression of miRNA-202-3p in serum. This method provides a good basis for further transition of miRNA-202-3p assessment into clinical testing in the future. However, the current research has some limitations. The most important limitation is that although serum miRNA-202-3p levels showed potential diagnostic value for type 1 g-NEN, the power of this miRNA as a diagnostic biomarker is moderate. Application of miRNA-202-3p in the clinic may require the integration of additional miRNAs to achieve higher diagnostic value, and this remains to be studied further. It should be noted that in some of the patients in our study, gastric tumours were present during the blood tests, whereas other patients had only neuroendocrine cell hyperplasia because the tumours had been removed. Therefore, further studies are needed to distinguish whether miRNA-202-3p is a biomarker of neuroendocrine cell hyperplasia or a specific biomarker of neuroendocrine tumours. In addition, the results of our experiments should be validated in a larger cohort to eliminate any errors that may have arisen due to the small cohort size. Future work could investigate additional potentially diagnostic miRNAs that could be used to develop a joint detection method such as Netest, an NEN detection algorithm featuring 51 genes [34].

## Conclusion

In conclusion, our results demonstrate increased serum miRNA-202-3p levels in patients with type 1 g-NEN, which has potential diagnostic value, with acceptable sensitivity and specificity. The results suggest that miRNA-202-3p has potential value as a biomarker of type 1 g-NEN and that it may provide a novel non-invasive strategy for diagnosing this disease.

## Data Availability

All data generated or analysed during this study are included in this published article. The experimental raw data and graphs are available from the corresponding author upon reasonable request.

## Abbreviations

g-NENs: Gastric neuroendocrine neoplasms
ECL cells: Enterochromaffin-like cells
CgA: Chromogranin A
miRNAs: MicroRNAs
RT: Reverse transcriptase
qRT-PCR: Quantitative real-time PCR
OLGIM: Operative link for gastric intestinal metaplasia assessment
ROC: Receiver operating characteristic
AUC: Area under the ROC curve

## References

[1] Modlin IM, Shapiro MD, Kidd M (2004). Siegfried Oberndorfer: origins and perspectives of carcinoid tumors. Hum Pathol.

[2] Bosman FT, Carneiro F, Hruban RH, Theise ND (2010). The 2010 WHO classification of tumors of the digestive system.

[3] Nagtegaal ID, Odze RD, Klimstra D, et al. The 2019 WHO classification of tumours of the digestive system. Histopathology, 2020. 10.1111/his.13975 PMC7003895 31433515

[4] Scarpa A, Fassan M, Borislav R, Claudio L, Capelli P (2013). Pathologist’s role in the management of gastroenteropancreatic neuroendocrine tumors (GEP-NETs). J Oncopathol.

[5] Ambros V (2004). The functions of animal microRNAs. Nature.

[6] Rushworth SA (2011). Targeting the oncogenic role of miRNA in human cancer using naturally occurring compounds. Br J Pharmacol.

[7] Endo Y, Toyama T, Takahashi S, Yoshimoto N, Iwasa M, Asano T, Fujii Y, Yamashita H (2013). miR-1290 and its potential targets are associated with characteristics of estrogen receptor α-positive breast cancer. Endocr Relat Cancer.

[8] Li SC, Khan M, Caplin M (2015). Somatostatin analogs treated small intestinal neuroendocrine tumor patients circulating MicroRNAs. PLoS ONE.

[9] Bowden M, Zhou CW, Zhang S (2017). Profiling of metastatic small intestine neuroendocrine tumors reveals characteristic miRNAs detectable in plasma. Oncotarget.

[10] Bi N, Cao J, Song Y (2014). A MicroRNA signature predicts survival in early stage small-cell lung cancer treated with surgery and adjuvant chemotherapy. PLoS ONE.

[11] Lloyd KA (2016). Gastrin-induced miR-222 promotes gastric tumor development by suppressing p27kip1. Oncotarget.

[12] Dou D, Shi Y-F, Liu Q (2018). Hsa-miRNA-202-3p, up-regulated in type 1 gastric neuroendocrine neoplasms, may target DUSP1. World J Gastroenterol.

[13] Nasir A, Sheikh U, Muhammad J, et al. Pathologic angiogenesis in neuroendocrine tumors. Neuroendocrine tumors: review of pathology, molecular and therapeutic advances. Springer New York, 2016.

[14] NCCN. Neuroendocrine Tumors, NCCN clinical practice guidelines in oncology (version 2. 2016) [EB/OL]. NCCN 2016. http://www.nccn.org, NET-4.

[15] Dasari A, Shen C, Halperin D, Zhao B, Zhou S, Xu Y, Shih T, Yao JC (2017). Trends in the incidence, prevalence, and survival outcomes in patients with neuroendocrine tumors in the United States. JAMA Oncol.

[16] Yao JC, Hassan M, Phan A, Dagohoy C, Leary C, Mares JE, Abdalla EK, Fleming JB, Vauthey JN, Rashid A, Evans DB (2008). One hundred years after “carcinoid”: epidemiology of and prognostic factors for neuroendocrine tumors in 35,825 cases in the United States. J Clin Oncol.

[17] Niederle MB, Hackl M, Kaserer K, Niederle B (2010). Gastroenteropancreatic neuroendocrine tumours: the current incidence and staging based on the WHO and European Neuroendocrine Tumour Society classification: an analysis based on prospectively collected parameters. Endocr Relat Cancer.

[18] Qiu X, Liu M, Liu Q (2017). Analysis of primary site and pathology on 903 patients with neuroendocrine neoplasms. Chin J Gastrointes Surg.

[19] Chen Y, Han D, Zhu J (2020). A prospective and retrospective clinical controlled observation of Chinese Herbal Decoction (SMLJ01) for type 1 gastric neuroendocrine tumors. Integr Cancer Ther.

[20] Robinson M (1999). Review article: current perspectives on hypergastrinaemia and enterochromaffin-like-cell hyperplasia. Aliment Pharmacol Ther.

[21] D'Elios MM, Bergman MP, Azzurri A, Amedei A, Benagiano M, De Pont JJ, Cianchi F, Vandenbroucke-Grauls CM, Romagnani S, Appelmelk BJ, Del Prete G (2001). H(+), K(+)-atpase (proton pump) is the target autoantigen of Th1-type cytotoxic T cells in autoimmune gastritis. Gastroenterology.

[22] Macukanović-Golubović L, Katić V, Rancić G, Milenović M, Marjanović G, Golubović Z (2007). Study on histogenesis of enterochromaffin-like carcinoid in autoimmune atrophic gastritis associated with pernicious anemia. Vojnosanit Pregl.

[23] He J, Li Y, Huang G, Cui M, Zhu J, Gu Y (2013). Analysis on biological information of has-miR-202. Beijing Biomed Eng.

[24] Zhang D, Li Y, Tian J, Zhang H, Wang S (2015). MiR-202 promotes endometriosis by regulating SOX6 expression. Int J Clin Exp Med.

[25] Yu J, Qiu X, Shen X, Shi W, Wu X, Gu G, Zhu B, Ju S (2014). MiR-202 expression concentration and its clinical significance in the serum of multiple myeloma patients. Ann Clin Biochem.

[26] Porkka KP, Pfeiffer MJ, Waltering KK, Vessella RL, Tammela TL, Visakorpi T (2007). MicroRNA expression profiling in prostate cancer. Cancer Res.

[27] Joosse SA, Müller V, Steinbach B, Pantel K, Schwarzenbach H (2014). Circulating cell-free cancer-testis MAGE-A RNA, BORIS RNA, let-7b and miR-202 in the blood of patients with breast cancer and benign breast diseases. Br J Cancer.

[28] Ueda T, Volinia S, Okumura H, Shimizu M, Taccioli C, Rossi S, Alder H, Liu CG, Oue N, Yasui W, Yoshida K, Sasaki H, Nomura S, Seto Y, Kaminishi M, Calin GA, Croce CM (2010). Relation between microRNA expression and progression and prognosis of gastric cancer: a microRNA expression analysis. Lancet Oncol.

[29] Fabbri M, Paone A, Calore F, Galli R, Gaudio E, Santhanam R, Lovat F, Fadda P, Mao C, Nuovo GJ (2012). MicroRNAs bind to Toll-like receptors to induce prometastatic inflammatory response. Proc Natl Acad Sci USA.

[30] Vickers KC, Palmisano BT, Shoucri BM, Shamburek RD, Remaley AT (2011). MicroRNAs are transported in plasma and delivered to recipient cells by high-density lipoproteins. Nat Cell Biol.

[31] Valadi H, Ekstrom K, Bossios A, Sjostrand M, Lee JJ, Lotvall JO (2007). Exosome-mediated transfer of mRNAs and microRNAs is a novel mechanism of genetic exchange between cells. Nat Cell Biol.

[32] Arroyo JD, Chevillet JR, Kroh EM, Ruf IK, Pritchard CC, Gibson DF, Mitchell PS, Bennett CF, Pogosova-Agadjanyan EL, Stirewalt DL (2011). Argonaute2 complexes carry a population of circulating microRNAs independent of vesicles in human plasma. Proc Natl Acad Sci USA.

[33] Schmittgen T D , Lee E J , Jiang J , et al. Real-time PCR quantification of precursor and mature microRNA. Methods, 2008, 44(1):0–38. 10.1016/j.ymeth.2007.09.006 PMC2663046 18158130

[34] Irvin M, Mark K, Anna M, Ignat D, Lisa B, Somer M, Kyung-Min C (2018). The NETest: the clinical utility of multigene blood analysis in the diagnosis and management of neuroendocrine tumors. Endocrinol Metab Clin N Am.

